# Ulcerative colitis combined with isolated superficially serrated adenoma: a Case Report

**DOI:** 10.3389/fonc.2025.1576166

**Published:** 2025-06-16

**Authors:** Hua Gao, Jinshan Nie, Lipin Zhang, Chen Gong

**Affiliations:** ^1^ Department of Gastroenterology, Taicang Affiliated Hospital of Soochow University, The First People’s Hospital of Taicang, Taicang, Jiangsu, China; ^2^ Department of Pathology, Taicang Affiliated Hospital of Soochow University, The First People’s Hospital of Taicang, Taicang, Jiangsu, China

**Keywords:** superficial serrated adenoma, ulcerative colitis, endoscopic resection, pathological diagnosis, case report

## Abstract

**Introduction:**

Superficially serrated adenoma (SuSA) is a recently identified type of serrated lesion characterized by a combination of adenomatous and serrated features. There are limited reports on SuSA currently, and its occurrence in conjunction with ulcerative colitis (UC) has not yet been documented.

**Case presentation:**

We present the case of a 38-year-old male with a 13-year history of ulcerative colitis (UC). During a routine follow-up colonoscopy, a superficial elevated lesion with a whitish surface was detected in the rectum, 10 cm from the anal verge. The lesion was removed en bloc via endoscopic submucosal dissection (ESD). Pathological analysis showed a combination of superficial serrated and vertically oriented adenomatoid glands. Immunohistochemistry indicated that the superficial epithelial cells exhibited serrated features and expressed CK20, while the middle and lower mucosal layers tested positive for Ki-67.

**Conclusion:**

Although some studies have explored SuSA, many aspects of the disease remain unclear. This is the first reported case of UC combined with SuSA. Future reports are essential to enhance understanding of the potential link between UC and SuSA.

## Introduction

Ulcerative colitis (UC) carries an increased risk of colorectal cancer due to the chronic nature of the disease, widespread inflammation and persistent inflammatory stimulation (1). Currently, the main oncogenic pathways of colorectal cancer include the classical adenoma-carcinoma sequence, the inflammation-cancer pathway, the *de novo* pathway and the serrated neoplasia pathway (2). Notably, the serrated neoplasia pathway accounts for 30% of sporadic colorectal cancer cases (3). According to the 2019 World Health Organization (WHO) classification, serrated lesions are now recognized as a new diagnostic category, “undetermined serrated adenoma”, alongside the established categories of hyperplastic polyp (HP), sessile serrated lesion (SSL) and traditional serrated adenoma (TSA) (4).

Superficially serrated adenoma (SuSA) is a new type of serrated lesion, first proposed by Japanese scholars Hashimoto et al. in 2018, characterized by its distinct pathological structure (5). The superficial epithelial cells exhibit a serrated microstructure, while proliferating cells are localized in the middle and lower layers. According to the recent WHO classification, SuSA can be categorized as an indeterminate serrated adenoma due to its ambiguous adenomatous and serrated morphology. SuSA is divided into two subtypes: isolated SuSA and TSA-associated SuSA, depending on whether it coexists with TSA. Notably, SuSA frequently exhibits concurrent KRAS mutations and RSPO fusions or overexpression. The RSPO fusions and overexpression, which lead to WNT signaling activation, are both common and specific to SuSA and TSA (6). SuSA is believed to be a potential precursor of microsatellite-stable colorectal cancer with KRAS mutations, posing some malignant risk and warranting careful monitoring (7). **Table 1** outlines the characteristics of HP, SSL, TSA, and SuSA.

**Table 1:** Characteristics of HP, SSL, TSA, and SuSA.

Characteristics	HP	SSL	TSA	SuSA
Endoscopic manifestations				
Endoscopic morphology	Pale, smooth	Pale, flat	Bright red, villous	White, flat
Size	Generally <5 mm	Generally >5 mm	Generally >5 mm	vary in size
Location	Distal colon	Proximal colon	Distal colon	Distal colon
Pit pattern	Type II	Type IIo	Type IV	Type II or Type IIIh
Histopathologic Characteristics				
Serration	Limited to top half of crypt	Top and bottom of crypt	Limited to top half of crypt	Superficial serration
Basal crypt architecture	Narrow, straight, tubular	Distorted, branching crypts dilated at base	Narrow, straight, tubular with protuberant villiform architecture	Straight tumor glands lacking serration in the middle to bottom layer
Nuclear features	Unremarkable	Small foci of pseudostratification	Elongated nuclei	Uniform elongated basal nuclei
Immunohistochemical analysis				
CK20	Expression in the upper layer	Negative expression	Weak expression	Expression in the upper layer
Ki-67 and MYC	Expression in the bottom layer	Negative expression	Expression in the upper layer	Expression in the middle to lower layer
β-Catenin	Positive expression	Negative expression	Negative expression	Positive expression
Mutation and gene expression				
KRAS mutations	Positive (goblet cell-rich HP)	Negative	Positive	Positive
BRAF mutations	Positive (microvesicular HP)	Positive	Positive	Negative

To date, there are few reports of UC combined with serrated lesions, but UC combined with SuSA has not been reported, which is clinically under-recognized. Here, we report a case of UC with isolated SuSA and review the related literature to summarize the clinical features and improve the detection rate.

## Case presentation

**Figure 1:**
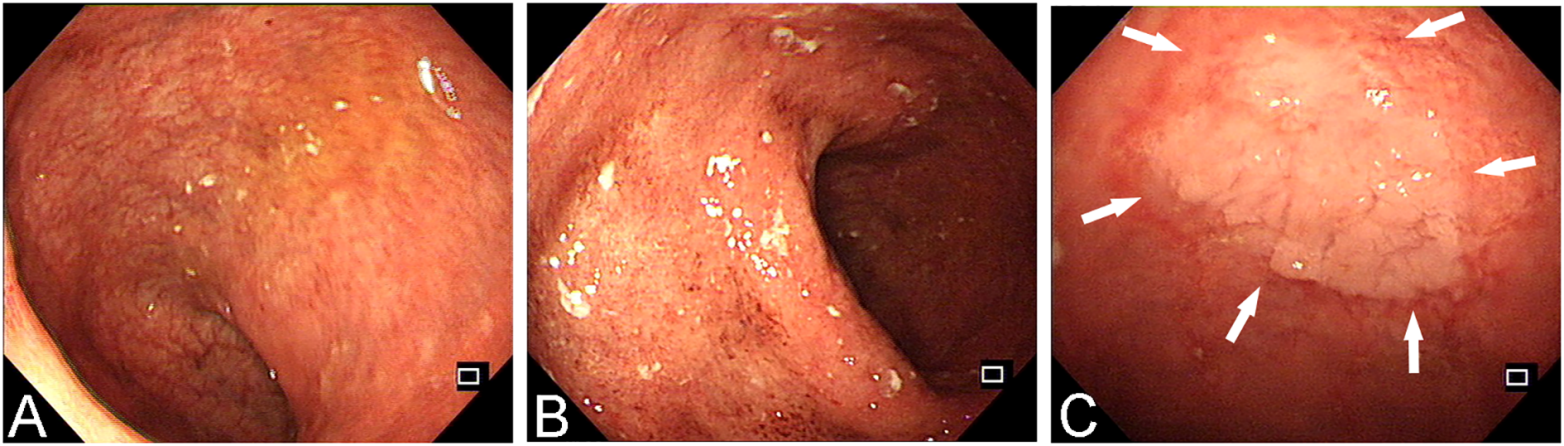
The endoscopic manifestations of a UC patient during a follow-up colonoscopy. **(A, B)** Enteroscopic examination reveals 20 cm of regressed intestinal mucosa, showing erosions, loss of vascular pattern, and erythema. These findings extend continuously to the rectum, with more pronounced abnormalities observed in the rectal region. **(C)** The white arrowheads shows a large, slightly elevated lesion in the rectum, characterized by a mildly lobulated surface.

**Figure 2:**
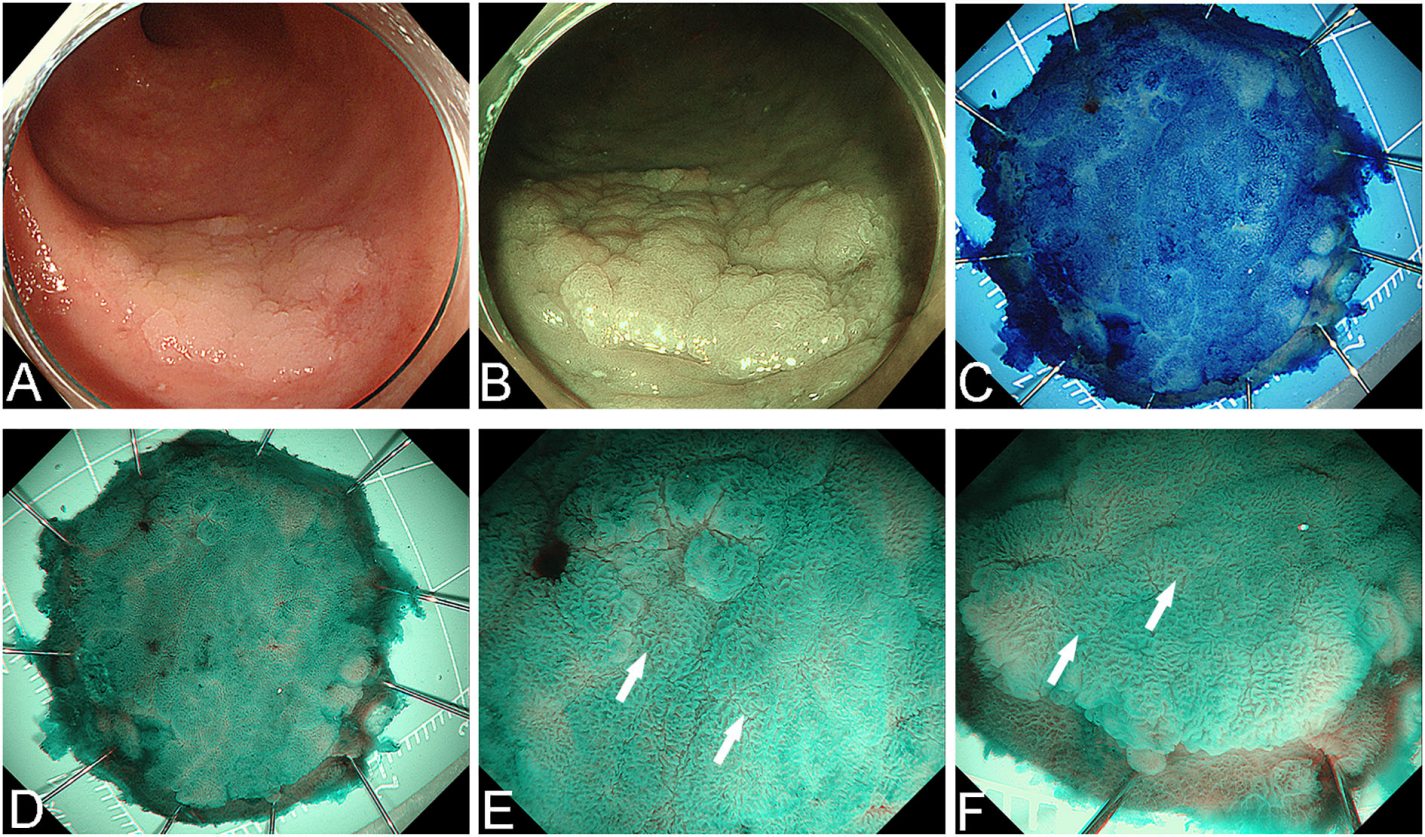
The lesion was removed en bloc by ESD. **(A)** White light endoscopy view before resection. **(B)** Narrow Band Imaging (NBI) view before resection. **(C)** Methylene blue staining of the postoperative specimen. **(D)** NBI observation of the specimen. **(E, F)** The surface pit pattern was predominantly classified as Type II (the white arrowheads mark the typical manifestations).

A 38-year-old male with a 13-year history of UC underwent a routine follow-up colonoscopy, marked erythema, erosions, mucosal fragility and loss of vascular texture were revealed in the rectosigmoid colon (E2, active, Mayo score 2). A superficially elevated lesion with a whitish surface, measuring approximately 2.1 x 2.0 cm, was detected in the rectum, 10 cm from the anal verge (**Figure 1**). Then, the lesion was removed en bloc by endoscopic submucosal dissection (ESD) (**Figures 2A, B**), and postoperative observation by endoscope revealed a predominant type II pit pattern (**Figures 2C–F**).

**Figure 3:**
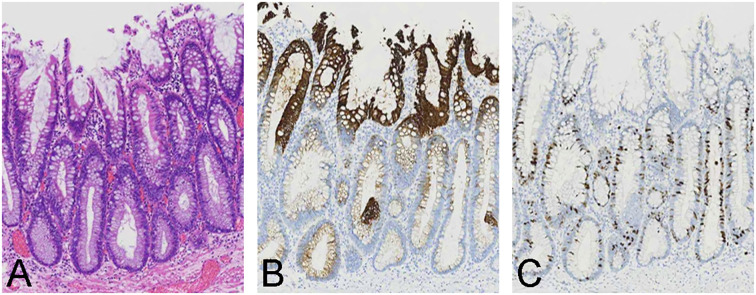
Histological and immunohistochemical findings of the postoperative specimen. **(A)** Pathological examination revealed a serrated surface, with intestinal heterotrophic glands present in the middle and lower layers. The nuclei were columnar, vertically arranged and associated with low-grade intraepithelial neoplasia. **(B)** CK20 expression was localized in the upper layer of the superficial serrated adenoma (SuSA). **(C)** Ki-67 expression was primarily observed in the middle and lower layers of the SuSA.

Histological examination of the specimen showed a serrated superficial layer with intestinal-type anisotropic hyperplastic glands and vertically arranged glands with low-grade intraepithelial neoplasia in the middle and lower layers. Immunohistochemical analysis demonstrated that the superficial epithelial cells had serrated features and expressed CK20, while Ki-67-positive cells were localized in the basal and middle layers of the glands in the flattened elevated area (**Figure 3**).

Given that there was no evidence of malignancy for this lesion, and in accordance with UC guidelines, patients who have a disease course exceeding 10 years are recommended to undergo colonoscopy once a year. The patient underwent a colonoscopy 11 months after ESD, during which no sign of recurrence was observed.

## Discussion

Patients with UC have an increased risk of colorectal cancer, which arises through different pathways. Approximately 15-35% of colorectal cancers develop via the serrated pathway (3), but the influence of UC on this pathway remains unclear. Studies on serrated lesions in patients with inflammatory bowel disease (IBD) are limited, mainly focusing on HP, SSL and TSA (8, 9). SuSA, a newly identified serrated lesion proposed by Hashimoto et al. in 2018, has not been previously reported in patients with UC. In this case, we reported an isolated SuSA in the context of active UC.

SuSA is a pre-cancerous lesion that follows the serrated neoplasia pathway (10). Hashimoto et al. (5) identified SuSA components in 15 out of 129 TSA through retrospective analysis. Additionally, Mizuguchi et al. (11) found only 24 cases of isolated SuSA among 21,000 endoscopically resected lesions between 2012 and 2018. They also identified 27 cases of TSA-associated SuSA among 184 TSA cases, suggesting that the prevalence of isolated SuSA was approximately 1 in 1,000, though further studies are needed to confirm the real-world prevalence.

SuSA lesions are predominantly located in the sigmoid colon or rectum. The endoscopic morphology of SuSA is white, flat mucosal lesion, with a well-defined, often irregular borders and lobulated structures appearance (11). Due to the consistently serrated surface microstructures, the pit pattern is typically classified as type II or type IIIh. Vascular morphology is generally not prominent in narrow-band imaging. SuSA lesions vary in size and appearance. Mizuguchi et al. (11) categorized isolated SuSA into three groups based on the lesion diameters: diminutive (1–5 mm), small (6–9 mm) and large (≥10 mm). Diminutive lesions tended to be round with a smooth, slightly elevated surface, while larger lesions often exhibited irregular margins and lobulated structures. In this case, the lesion matched the typical endoscopic appearance of a large isolated SuSA. Sato et al. (12) reported that TSA-associated SuSA typically had a double-layered elevated appearance, with a white base (SuSA) and a red upper portion (TSA). The SuSA component showed a type II or IIIh pit pattern, whereas the TSA component exhibited a type IVh pit pattern, highlighting the differences between isolated SuSA and TSA-associated SuSA.

Histological examination of SuSA showed a combination of superficial serrated and vertically oriented adenomatoid glands. Immunohistochemistry showed CK20 positivity in the superficial epithelial cells and Ki-67 positivity in the basal and middle layers, consistent with the typical mixed adenomatous and serrated features of SuSA. The Ki-67 expression in the middle and lower layers, indicating proliferative cell colonization, distinguishes SuSA from tubular adenomas. In contrast, Sato et al. (12) described a TSA-associated SuSA with a clear boundary between the TSA and SuSA components. In their case, the SuSA component consisted of serrated adenomatous glands with serrations confined to the superficial epithelium and crypt orifices, while the TSA component had dense glandular proliferation with pseudocomplex nuclei.

The first step in the development of serrated polyps via the serrated pathway involves mutations in genes that regulate the mitogen-activated protein kinase (MAPK) pathway, such as KRAS or BRAF in some cases. These mutations affect downstream molecules like EGFR, which play critical roles in cell proliferation, differentiation and survival. Most studies on SuSA were focused on TSA-associated cases. SuSA commonly exhibits concurrent KRAS mutations and RSPO fusion/overexpression, supporting its classification as a serrated lesion. KRAS mutations are consistent in both SuSA and TSA components, suggesting some malignant potential (7, 11). In this case of isolated SuSA, typical endoscopic and histopathologic features were present, but further molecular testing was not conducted due to patient preferences. More research is needed to explore the molecular mechanisms underlying isolated SuSA, particularly whether UC-related inflammation alters these mechanisms.

In summary, this case involved a young man with UC diagnosed with SuSA at the rectum, characterized by a white surface and a predominantly type II pit pattern. To the best of our knowledge, this is the first reported case of UC combined with SuSA. The potential link between UC and SuSA requires further investigation.

## Data Availability

The raw data supporting the conclusions of this article will be made available by the authors, without undue reservation.
